# Review: Application of Artificial Intelligence in Phenomics

**DOI:** 10.3390/s21134363

**Published:** 2021-06-25

**Authors:** Shona Nabwire, Hyun-Kwon Suh, Moon S. Kim, Insuck Baek, Byoung-Kwan Cho

**Affiliations:** 1 Department of Biosystems Engineering, Chungnam National University, Daejeon 34134, Korea; nabwireshona@o.cnu.ac.kr; 2 Department of Life Resources Industry, Dong-A University, Busan 49315, Korea; 3 Environmental Microbial and Food Safety Laboratory, Agricultural Research Service, United States Department of Agriculture, Powder Mill Road, BARC-East, Bldg 303, Beltsville, MD 20705, USA; moon.kim@usda.gov (M.S.K.); insuck.baek@usda.gov (I.B.); 4 Department of Smart Agriculture System, Chungnam National University, Daejeon 34134, Korea

**Keywords:** artificial intelligence, deep learning, plant phenomics, field phenotyping, high throughput phenotyping, image-based phenotyping

## Abstract

Plant phenomics has been rapidly advancing over the past few years. This advancement is attributed to the increased innovation and availability of new technologies which can enable the high-throughput phenotyping of complex plant traits. The application of artificial intelligence in various domains of science has also grown exponentially in recent years. Notably, the computer vision, machine learning, and deep learning aspects of artificial intelligence have been successfully integrated into non-invasive imaging techniques. This integration is gradually improving the efficiency of data collection and analysis through the application of machine and deep learning for robust image analysis. In addition, artificial intelligence has fostered the development of software and tools applied in field phenotyping for data collection and management. These include open-source devices and tools which are enabling community driven research and data-sharing, thereby availing the large amounts of data required for the accurate study of phenotypes. This paper reviews more than one hundred current state-of-the-art papers concerning AI-applied plant phenotyping published between 2010 and 2020. It provides an overview of current phenotyping technologies and the ongoing integration of artificial intelligence into plant phenotyping. Lastly, the limitations of the current approaches/methods and future directions are discussed.

## 1. Introduction

According to data from the United Nations, the world population is expected to grow to nine billion by 2050 [1]. With the increasing need for food production to match the projected population growth in order to prevent food insecurity, plant phenotyping is now at the forefront of plant breeding as compared to genotyping [2]. Plant phenotyping is defined as the assessment of complex traits such as growth, development, tolerance, resistance, architecture, physiology, ecology, yield, and the basic measurement of individual quantitative parameters that form the basis for complex trait assessment [2]. Scientists are increasingly interested in the use of phenomic-level data to aid in the correlation between genomics and the variation in crop yields and plant health [3,4,5]. In this way, plant phenotyping has become an important aspect of crop improvement, availing data to assess traits for variety selection in order to identify desirable traits and eliminate undesirable traits during the evaluation of plant populations [6]. Plant phenotyping has improved progressively over the past 30 years, although obtaining satisfactory phenotypic data for complex traits such as stress tolerance and yield potential remains challenging [7]. The large data required for the effective study of phenotypes has led to the development and use of high-throughput phenotyping technologies to enable the characterization of large numbers of plants at a fraction of the time, cost, and labor of previously used traditional techniques. Traditional techniques previously required destructive measurements whereby crops were harvested at particular growth stages in order to carry out genetic testing and the mapping of plant traits [8]. Since crop breeding programs require repeated experimental trials in order to ascertain which traits are of interest, the process was slow, costly and significantly lagging behind the DNA sequencing technologies which are necessary for crop improvement [9].

High-throughput phenotyping has been fostered by non-invasive imaging techniques, which have enabled the visualization of plant cell structures on a wider scale. As these imaging technologies develop, images carry more useful extractable information that supports biological interpretations of plant growth [10,11]. These techniques include thermal imaging [12], chlorophyll fluorescence [13,14], digital imaging [15], and spectroscopic imaging [16].

High-throughput phenotyping techniques are currently being used to enable data acquisition in both laboratory and field settings. They are being employed at the levels of data collection, data management, and analysis. They include imaging sensors, growth chambers, data management and analysis software, etc. [7,17,18,19,20]. The integration of artificial intelligence into these technologies has contributed to the development of the non-invasive imaging aspect of phenomics. Artificial intelligence (AI) technologies in the form of computer vision and machine learning are increasingly being used to acquire and analyze plant image data. Computer vision systems process digital images of plants to detect specific attributes for object recognition purposes [21]. Machine learning employs various tools and approaches to ‘learn’ from large collections of crop phenotypes in order to classify unique data, identify new patterns and features, and predict novel trends [22,23]. Recent advancements in deep learning, a subset of machine learning, have provided promising results for real-time image analysis. Deep learning is a machine learning approach that takes advantage of the large plant datasets available and uses them to carry out image analysis using convolutional neural networks [24]. In field phenotyping, AI is being applied in field equipment for ground and obstacle detection, the detection of plants and weeds during data collection, and the stable remote control of the equipment [25]. Although the field phenotyping applications of AI are in relative infancy compared to laboratory phenotyping, their growth is notable because they provide the phenotypic data of plants in their natural environment.

In addition to image analysis, AI applications that are widely used in other domains of science are now being integrated into the phenomics data management pipeline. Cyberinfrastructure (CI), a research environment that provides linkages between researchers, data storage, and computing systems using high-performance networks has been applied widely in the environmental sciences [26,27,28]. CI is now being applied to phenomics in order to facilitate collaboration among researchers [29]. Open-source devices and tools represent another fast developing application of AI technologies [30]. In phenomics, these tools are addressing the challenges of expensive phenotyping equipment and proprietary or incompatible data formats. The growth of these applications of AI has expanded the field of phenomics with industry companies investing in the manufacture and distribution of phenotyping technologies alongside government-funded agricultural institutions.

This review begins by considering the broader area of artificial intelligence and its integration and application in phenomics through machine learning and deep learning. Digital, fluorescence, spectroscopic, thermography, and tomography imaging, and the integration of artificial intelligence into their individual data management are highlighted. Thereafter, additional applications of AI such as cyberinfrastructure and open-source devices and tools are discussed. The current utilization of phenotyping technologies for field phenotyping, which is increasingly gaining ground over phenotyping in controlled environments, is then discussed briefly, highlighting their cross applicability with artificial intelligence.

## 2. Artificial Intelligence

Artificial Intelligence (AI) is widely referred to as the simulation of human intelligence in machines which are programmed to think like humans and mimic their actions. It is applied when referring to machines that exhibit traits associated with the human mind [31]. The recent emergence and growth of AI in academia has presented an opportunity, as well as a threat, for various domains in the sciences. Whereas some predict that the application of AI through robotics could lead to technological unemployment, it has enabled science to extend into areas previously unexplored and provided ease of execution, for example, in medical diagnostics [32].

**Figure 1:**
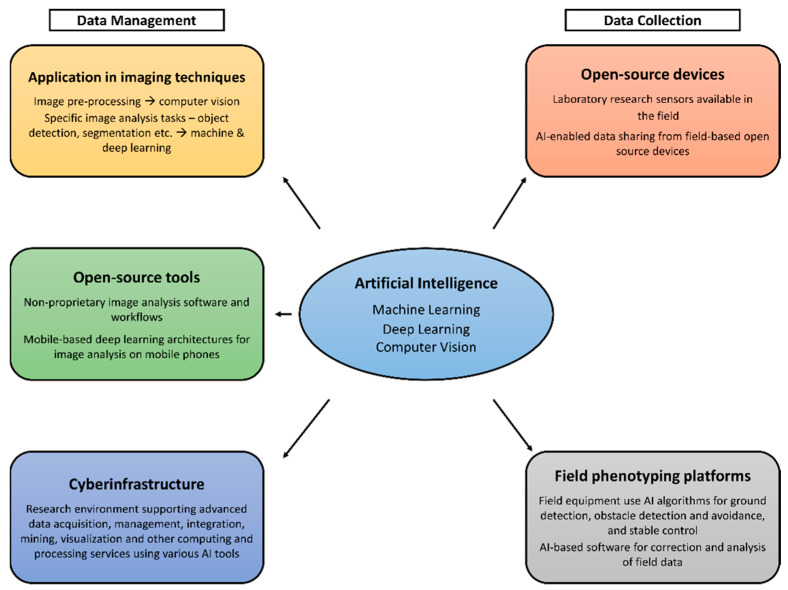
Workflow illustrating application of AI in phenomics.

In order to effectively program machines for desired tasks, AI methods call for large repositories of data. The algorithms used in AI methods need large sets of data for training to facilitate decision support by enhancing early detection and thereby improving decision-making [33]. The data acquisition process in non-destructive phenomics involves integrating the data from instruments/sensors (i.e., digital cameras and spectrometers), usually equipped with their individual, proprietary communication protocols, into the AI algorithms. The sensor outputs often require conversion to compatible digital formats before analysis [7]. Phenomic data management therefore involves three critical components where artificial intelligence is applied: algorithms and programs to convert the sensory data into phenotypic information; model development to understand the genotype–phenotype relationships with environmental interactions; and the management of databases to allow for the sharing of information and resources [6]. The main aspects of AI, machine learning, deep learning, and computer vision have been applied thus far to a recognizable extent in phenomics (illustrated in Figure 1). Other areas of application are cyberinfrastructure and open-source devices and tools, which will subsequently be discussed in detail.

### 2.1. Machine Learning

Since the 1970s, AI research has been focused on machine learning. Statistical machine learning frameworks and models such as Perceptron, support vector machines, and Bayesian networks have been designed. However, no single model works best for all tasks. It is still challenging to determine the best model for a given problem [34]. According to Roscher et al. 2020 [35], the rise and success of neural networks, coupled with the abundance of data and high-level computational and data processing infrastructure, has led to the comprehensive utilization of machine learning (ML) models and algorithms despite the challenge of model determinations for a given task.

The utilization of a range of imaging techniques for nondestructive phenotyping has influenced the development of high-throughput phenotyping (HTP), whereby multiple imaging sensors collect plant data in near-real-time platforms. These sensors have the capacity to collect large volumes of data which has, in turn, made phenomics a big data problem well suited for the application of ML. The analysis and interpretation of these large datasets are quite challenging, but ML algorithms provide an approach for faster, efficient, and better data analytics than traditional processing methods. Traditional processing methods include probability theory, decision theory, optimization, and statistics. ML tools leverage these processing methods to extract patterns and features from these large amounts of data in order to enable feature identification in particular complex tasks such as stress phenotyping [36].

According to Rahaman et al. 2019 [37], one of the advantages of using ML approaches in plant phenotyping is their ability to search large datasets and discover patterns by simultaneously looking at a combination of features (compared to analyzing each feature separately). This was previously a challenge because of the high dimensionality of plant images and their large quantity, making them difficult to analyze through traditional processing methods [36]. Machine learning methods have thus far been successfully applied in the identification and classification of plant diseases [38,39] and plant organ segmentation, among other tasks as shown in Table 1 below. This has been achieved by supervised learning, where the algorithms can identify diseased plants after being trained with sample images from large datasets. However, this approach prevents the search for novel and unexpected phenotypic traits that would otherwise be discovered by the less accurate unsupervised ML [40].

### 2.2. Deep Learning

Deep learning is a rapidly advancing subset of machine learning tools that has created a paradigm shift in image-based plant phenotyping. It is efficient in the discovery of complex structures in high-dimensional data and is thus applicable to a range of scientific research tasks [24]. The plant images collected using the various sensors have a wide variability, making the use of some machine learning techniques challenging [47]. While traditional machine learning involves trial-and-error steps in the feature extraction process of images, deep learning tools have enabled the creation of more reliable workflows for feature identification. They employ an automatic hierarchical feature extraction process using a large bank of non-linear filters before carrying out decision-making, such as classification. Deep learning approaches have multiple hidden layers in the network, with each layer performing a simple operation on the images in succession which increases their discrimination and prediction ability [48]. A wide range of deep learning architectures has been used in plant phenotyping by a process called transfer learning. This involves the use of a network that was pre-trained on a large dataset somewhat similar to the one under investigation and retraining it with weights for the new dataset. Table 2 details some deep learning architectures that have been applied using transfer learning for phenotyping.

Even with the current influx of deep learning architectures, they still face a few challenges in their integration with agricultural applications. There is still limited availability of publicly available annotated agricultural data, which reduces the possibility of obtaining high-performance feature extraction models through transfer learning. In addition, many agricultural image data have high levels of occlusion (especially plant leaves and background noise), leading to higher likelihoods of error from confusing objects of interest with the background. This is partly due to the environmental variations (e.g., cloudy sky, windy weather for field data collection) that significantly impact the images and make them harder to work with. Similarly, data samples are also sensitive to imaging angles, field terrain and conditions, and variations within plant genotypes. Hence, the robustness and adaptability requirements are significantly high for the deep learning models built for agricultural applications [55].

Thus far, deep learning architectures in phenotyping have been used in leaf counting [56], the classification of plant morphology [57], plant recognition and identification [55], root and shoot feature identification [48], and plant stress identification and classification [58]. A few reported applications of machine learning or deep learning for stress prediction and quantification provide great opportunities for new research efforts of plant scientists. A key challenge to overcome is that the underlying processes for linking the inputs to the outputs are too complex to model mathematically.

## 3. Application of Artificial Intelligence in Phenotyping Technologies

### 3.1. Imaging Techniques

Traditionally, the measurement of observable plant traits has been conducted by destructive sampling followed by laboratory determinations to characterize phenotypes based on their genetic functions. Due to technological advancement in AI, imaging techniques (overview in Table 3) have emerged as important tools for non-destructive sampling, allowing image capture, data processing, and analysis to determine observable plant traits. According to Houle et al. 2010 [5], “imaging is ideal for phenomic studies because of the availability of many technologies that span molecular to organismal spatial scales, the intensive nature of the characterization, and the applicability of generic segmentation techniques to data.” Spatial or temporal data of many phenotype classes such as morphology and geometric features, behavior, physiological state, and locations of proteins and metabolites can be captured in intensive detail by imaging. For that reason, imaging techniques have allowed for high-throughput screening and real-time image analysis of physiological changes in plant populations. At the laboratory scale, the different imaging methods are tested individually. One, or a combination of the best-suited methods for crop surveillance, are then used both in controlled and field environments [5,59].

**Figure 2:**
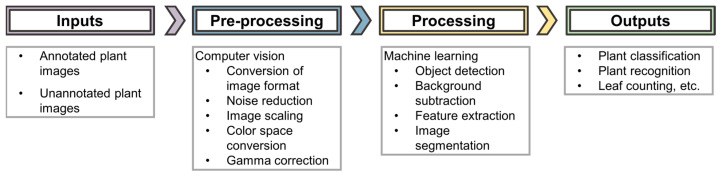
AI workflow for image analysis.

Computer vision is the major aspect of artificial intelligence that is applied in these imaging techniques. Zhuang et al. 2017 [34], comprehensively state that “computer vision aims to bring together factors derived from multiple research areas such as image processing and statistical learning to simulate human perception capability using the power of computational modeling of the visual domain.” Computer vision uses machine learning to recognize patterns and extract information from the images. The computer vision workflow (shown in Figure 2) carries out visual tasks ranging from pre-processing (e.g., conversion of image formats, color space conversions etc.) to object detection applied in ML algorithms, resulting in the comprehension of image understanding in a human-like way [34]. Computer vision transforms the images so that they can be applied to an AI system. This process has high computational demands, especially when working with images of varying formats from a range of sensors, as is the case in phenotyping imaging techniques [32]. A few of the different imaging techniques are discussed below.

#### 3.1.1. Digital/RGB Imaging

Digital imaging is the lowest costing and easiest to use imaging technique. Its images comprise pixels from a combination of the red, green, and blue (RGB) color channels. RGB camera sensors are sensitive to light in the visible spectral range (400–700 nm). Within this range, they are able to extract images that can be used to depict some significant physiological changes in a biological sample. RGB/digital imaging depends on the color variation of different biological samples and has significantly contributed to various plant phenotyping aspects [75,76]. It tracks the color changes and directly helps in monitoring the status of plant developmental stage, morphology, biomass, health, yield traits, and stress response mechanisms. These mechanisms can be measured rapidly and accurately for large populations. Digital imaging also provides information on the size and color of plants, which enables the quantification of plant deterioration arising from, for example, nutrient deficiencies or pathogen infections, etc. Using a combination of careful image capture, image analysis, and color classification, it is possible to follow the progression of lesions from infections and deficiencies over time quantitatively [8].

Advances in hardware and software for digital image processing have motivated the development of machine vision systems providing for the expeditious analysis of RGB images. Being relatively the simplest and most widely used technique has served as an advantage, as many machine learning techniques and deep learning architecture can be applied effectively to RGB images. These techniques have been applied in the identification of plant growth stage [41], classification of plant images [43,44,45,46], disease detection and classification [38,52,53,65], identification of biotic and abiotic stress [49,58], weed detection [50,51], detection of flowering times [57], and leaf counting [54,55,56]. Although the extraction of useful phenotyping features from 2D digital images has been successful, the expansion in computer vision has led to an exploration of the applications of 3D imaging. This has been achieved using stereo-vision, where two identical RGB cameras are used to capture images in a setup similar to the operation of the human eyes. These images are then used to reconstruct a 3D model of the plant for analysis using stereo-matching algorithms [60,77]. Approaches using multiple images from more than two RGB cameras placed at different viewing angles have been successfully used for larger plants with a higher degree of occlusion thereby expanding the applications of digital imaging [78].

#### 3.1.2. Spectroscopy

Spectroscopic imaging is a widely used imaging technique that has been used to predict many properties of large plant populations [5]. It consists of multispectral and hyperspectral imaging. In multispectral imaging, the images are captured in wavelengths between visible and near-infrared, consisting of up to fifteen spectral bands, whereas in hyperspectral imaging, hundreds of continuous spectral wavebands are available. Previous phenotyping studies have shown that spectroscopy can be used to monitor plant photosynthetic pigment composition, assess water status, and detect abiotic or biotic plant stresses [79]. During plant development, varying growth conditions induce changes in surface and internal leaf structure, modifying the reflection of light from plant leaves or canopies. These changes can be visualized by spectroscopy, either in the visible spectrum or near-infrared wavelengths undetectable by the human eye (0.7–1.3 mm) [18]. The application of spectroscopy is therefore important for the field monitoring of plant traits arising from gene expression in response to environmental factors [20].

Hyperspectral imaging has thus far been successfully applied in both controlled environments (i.e., greenhouses and growth chambers) and field environments [80]. However, a major limitation to the utility of hyperspectral data in field phenotyping, besides the cost of the equipment, is the variability in environmental conditions during measurements. Spectrometers are highly sensitive and rely on solar radiation as a light source in the field, and this leads to difficulty in the analysis of images due to cloud cover, shadows caused by phenotyping platforms, and changes in solar angle during the photo period [8,80]. Another challenge in hyperspectral data analysis is data redundancy due to the continuous nature of wavelengths and their similarity. This has been alleviated by the selection of effective wavelengths using algorithms such as the successive projections algorithm (SPA), genetic algorithm (GA), the Monte-Carlo uninformative variable elimination (MC-UVE), and boosted regression tree (BRT) which is also a ML technique [81,82,83].

Although these difficulties can potentially be remedied by applying robust computer vision algorithms, hyperspectral images have only been successfully applied to machine learning algorithms and not to faster and more advanced deep learning algorithms. Nonetheless, hyperspectral imaging allows for a wide variety of stresses to be detected and continues to be a promising way to detect specific signatures for a particular stressor [18].

#### 3.1.3. Thermography

Thermography, also known as thermal imaging, is a technique that detects infra-red radiation from an object and creates an image based on it. Thermographic cameras detect infrared radiation (9000−14,000 nanometers) in the electromagnetic spectrum and create images based off of it [84]. Thermography has been used in plant research to monitor transpiration and canopy temperature. Transpiration is linked with nutrient uptake by the roots and, ultimately, with crop productivity. However, it also reflects water use efficiency. Canopy temperature has been widely used to infer crop water use, photosynthesis, and, in some cases, to predict yield. In breeding programs aimed at selecting plants based on water use efficiency, thermography improves the speed and effectiveness of monitoring transpiration [18]. It has also been used in the field as a remote sensing tool to capture canopy temperature data for a large number of plots using microbolometer-based thermal imaging mounted on field phenotyping platforms above the crop using helium balloons or manned aircraft [8]. Despite the inability of thermography to detect pre-symptomatic changes in leaves, it can detect changes in leaf thickness [18]. This allows for the visualization and monitoring of internal structural heterogeneity resulting from stresses or infections.

In phenotyping, thermography is used in combination with other imaging techniques for effective diagnostics [85]. Photogrammetry algorithms such as structure-from-motion have been applied to thermographic images [86] collected in field environments without much success. Currently, the data collected from the images is analyzed using standard equations and ML statistical methods such as probability theory, decision theory and classifiers [87]. Thermography has a range of applications from medical diagnostics to metal defect detection in industries where deep learning algorithms have been applied to thermal images [88].

#### 3.1.4. Fluorescence

Fluorescence imaging, also known as fluorescence spectroscopy, is used as a measurement technique for photosynthetic function under stresses such as drought and infections by detecting light emitted after the plant has been exposed to a specific wavelength of light. These stresses have adverse effects that lead to a decrease in photosynthesis which, in turn, limits crop yield. Chlorophyll fluorescence imaging has enabled the early visualization of viral and fungal infections due to its ability to achieve high resolutions. It has also been used in studies to determine plant leaf area [18]. For the rapid screening of plant populations, portable fluorometers are being used to obtain average measurements of whole plants or leaves at the same developmental stage. There is potential for portable fluorescence imaging to be used for the field-scale assessment of infections, even for those that leave no visible trace [8,18,67]. Fluorescence imaging is usually used in combination with hyperspectral imaging, and image data extracted using this technique has been successfully applied to algorithms based on AI methods, such as neural networks, for analysis [89].

#### 3.1.5. Tomography

X-ray computed tomography (X-ray CT) is a technology that uses computer-processed X-rays to produce tomographic images of specific areas of scanned objects. It can generate a 3D image of the inside of an object from an extensive series of 2D radiographic images taken around a single axis of rotation [76]. X-ray CT imaging technology has been used for several applications in plant phenotyping. It has been applied in the observation of root growth because of its ability to capture the intricacies of the edaphic environment with high spatial resolutions [90,91]. X-ray CT has also been used in the high-throughput measurement of rice tillers to determine grain yield. It was applied as the preferred imaging technique for the rice tiller study because of the tendency of rice tillers to overlap and, hence, not be easily detectable by digital imaging [92]. According to Li et al. 2014 [76], however, “tomographic imaging remains low throughput, and its image segmentation and reconstruction need to be further improved to enable high throughput plant phenotyping.” Although this technology is effective in the early detection of plant stress symptoms, its effectiveness is further improved by combined use with other imaging techniques. The simultaneous use of CT and positron emission tomography (PET) has the potential to be used to provide insight into the effect of abiotic stress in particular [71,76]. Additionally, to provide satisfactory resolutions, X-ray CT requires small pot sizes and controlled environments, making it unsuitable for field applications. For morphological root phenotyping tasks, X-ray CT has been applied in the identification of root tips and root-soil segmentation tasks using machine learning [93]. Despite the minimal use of tomography in phenotyping, its application in the medical field positions it as a powerful technique that, coupled with AI algorithms (and particularly CNNs), is beneficial in diagnostics [94].

### 3.2. Cyberinfrastructure

**Figure 3:**
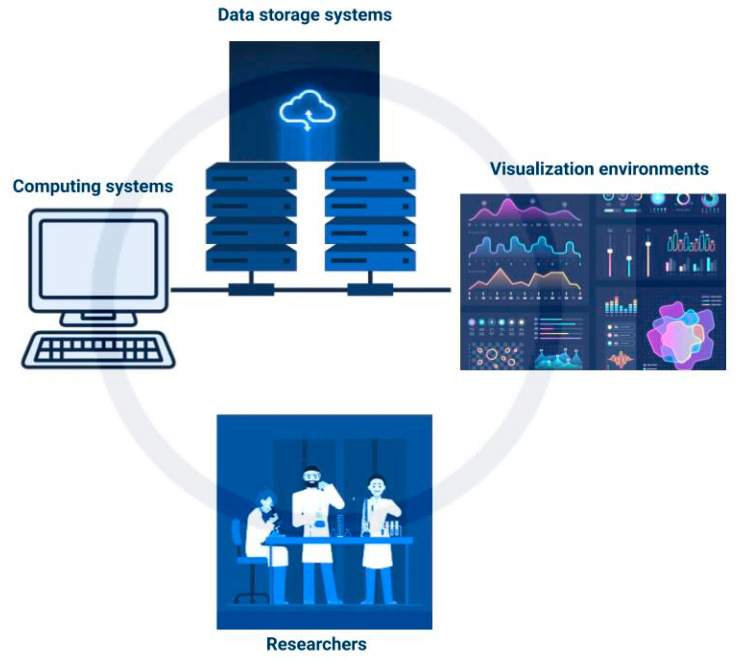
Simplified schematic of cyberinfrastructure.

Cyberinfrastructure (CI) is described by Atkins et al. 2003 [95] as a “research environment that supports advanced data acquisition, storage, management, integration, mining, visualization and other computing and processing services distributed over the internet beyond the scope of a single institution.” It consists of computing systems, data storage systems, advanced instruments and data repositories, visualization environments, and people (shown in Figure 3) linked together by software and high-performance networks [27]. This enhances the efficiency of research and productivity in the use of resources. Some CI systems can provide for in-field data analysis to point out errors in data collection that can be rectified and identify further areas of interest for data collection.

CI has gained more interest in recent years because of the growth in quantities of data collected in science, interdisciplinarity in research, the establishment of a range of locations around the world where cutting-edge research is performed, and the spread of advanced technologies [96]. Due to the various components required, CI systems are expensive, with the cost of a supercomputer alone being upwards of US$90 million. For this reason, some organizations that can invest in this infrastructure offer it as a service at a fee to researchers. For example, the University of Illinois at Chicago provides various cost models for access to their infrastructure [97].

CI has been applied in scientific disciplines ranging from biomedical to geospatial and environmental sciences. One such project is the distributed CI called the Function Biomedical Informatics Research Network (FBIRN), a large-scale project in the area of biomedical research funded by the U.S. National Institutes of Health (NIH) [96]. CI has also been applied in geospatial research with a range of initiatives under the National Spatial Data Infrastructure (NSDI). The NSDI focuses on spatial data collection from government and private sources, integration and sharing through its GeoPlatform (https://www.geo-platform.gov/, accessed on 26 June 2021) [98]. In the geospatial domain, one example is the Data Observation Network for Earth (DataONE), which is a CI platform for integrative biological and environmental research. It is designed to provide an underlying infrastructure that facilitates data preservation and re-use for research with an initial focus on remote-sensed data for the biological and environmental sciences [99]. Because of the complexities in developing and setting up a CI, Wang et al. 2013 [100] put forward the Cyberaide Creative service, which uses virtual machine technologies to create a common platform separate from the hardware and software and then deploys a cyberinfrastructure for its users. This allows for end-users to specify the necessary resource requirements and have them immediately deployed without needing to understand their configuration and basic infrastructures.

In plant phenotyping, a case for the use of CI has been made similarly in that non-invasive high throughput phenotyping technologies collect large amounts of plant data. Analysis methods for this data using AI are being developed but face the challenge of integrating datasets and the poor scalability of these tools [29]. The large amounts of data generated by HTP platforms need to be efficiently archived and retrieved for analysis. Researchers affiliated with the United States National Science Foundation (NSF) have developed a form of CI called iPlant that incorporates artificial intelligence technologies to store and process plant data gathered from the various HTP platforms. This CI platform provides tools for data analysis and storage with high-performance computing to access and analyze the data. It also has methods for the integration of tools and datasets [29].

In order to support both genotyping and phenotyping, iPlant uses the BISQUE (Bio-Image Semantic Query User Environment) [101] software system. Its main functionality is image analysis which it supports using its five core services: image storage and management, metadata management and query, analysis execution, and client presentation. Its design is flexible enough to support the range of variability in image analysis workflows between research labs. The plant-oriented version of BISQUE, PhytoBISQUE, provides an application programming interface integrated with iPlant to develop and deploy new algorithms, facilitating collaboration among researchers [29].

### 3.3. Open-Source Devices and Tools

Open-source is a term generally used to refer to tools or software that, as stated by Aksulu & Wade, 2010 [30], “allows for the modification of source code, is freely distributed, is technologically neutral, and grants free subsidiary licensing rights.” Characteristically, Open-Source Systems (OSS) are voluntary and collaborative in nature and their lifespan lasts as long as there is an individual willing and able to maintain the system [102]. Few traditional operation constraints such as scope, time, and cost factors affect these systems, and they have the added advantage of enhancing the skills of the people involved while producing tangible cost-effective technology output [30]. Some OSS development teams take advantage of crowdsourcing which widens the scope and quality of ideas and reduces project cycle time [103].

In phenomics, new crop management strategies require the co-analyses of both sensor data on crop status and related environmental and genetic metadata. Unfortunately, this data is mostly restricted to larger well-funded agricultural institutions since the instruments for data collection are expensive. The available phenotyping instruments output data that is challenging to interpret because of proprietary or incompatible formats. Many techniques that are being applied are ready-made off-the-shelf software packages that do not have specific algorithms for this data interpretation. Phenotyping researchers, therefore, have to address the challenges of data interpretation and data sharing alongside limited access to instrumentation (especially that which is well suited for field phenotyping). Open-source tools and devices represent a promising approach for addressing these challenges. Those being applied in phenomics are more accessible and easy to use while providing a connection to a community of users with broader support and continuous improvement [104,105]. One such open-source device is the MultispeQ device (PhotosynQ, East Lansing, MI, USA), an inexpensive device linked through the PhotosynQ platform (https://www.photosynq.com, accessed on 1 June 2021) to communities of researchers, providing useful data on plant performance. The MultispeQ device is rugged and field deployable, open-source, and expandable to incorporate new sensors and techniques. The PhotosynQ platform connects the MultispeQ instrument to the community of researchers, breeders, and citizen scientists to foster field-based and community-driven phenotyping [105].

An open-source prediction approach called Dirichlet-aggregation regression (DAR) was put forward by Bauckhage and Kersting, 2013 [104], to address the challenge of manual data labeling and running supervised classification algorithms on hyperspectral data. Hyperspectral cameras record a spectrum of several hundred wave-lengths ranging from approximately 300 nm to 2500 nm, which poses a significant challenge of data handling in hyperspectral image analysis. Therefore, working with hyperspectral data requires algorithms and architecture that can cope with massive amounts of data. Their research shows that DAR can predict the level of drought stress of plants effectively and before it becomes visible to the human eye.

Open-source software and platforms have also been developed that simplify the computer vision image management pipeline. One such tool is the PlantCV image analysis software package. It is used to build workflows that can be used to extract data from images and sensors, and it employs various computational tools in python that are extendable, depending on the required image analysis task, in order to provide data scientists and biologists with a common interface [106]. It deploys additional open-source tools such as LabelImg for image annotation [107]. PlantCV’s image processing library has been applied in Deep Plant Phenomics which is an open-source software platform that implements deep convolutional neural networks for plant phenotyping [108]. Deep Plant Phenomics provides an image processing pipeline that has been used for complex non-linear phenotyping tasks such as leaf counting, mutant classification, and age regression in *Arabidopsis*.

Another group of open-source tools that have been applied in phenomics are the MobileNet deep learning architectures. MobileNet architectures are convolutional neural networks (CNNs) with reduced complexity and model size and are suited to devices with low computational power such as mobile phones. MobileNets optimize for latency resulting from low computing power by providing small networks with substantial accuracy that can be used in real-world applications [109]. One example is the MobileNetV2 which is built to be used for classification, detection, and segmentation of images. It uses ReLU6 non-linearity, which is suited to low-precision computation [110]. These are promising for use on mobile phones which are widely accessible and commonplace to be potentially used for field phenotyping. MobileNets employ TensorFlow Lite, an open-source deep learning framework used to deploy machine learning models on mobile devices [111].

Lighting problems in outdoor settings have the potential to affect open-source tools and the performance of CNNs. There is, therefore, a need to test and train the networks on plant images collected in the field. A mobile-based CNN model was used for plant disease diagnosis, and the problem of inconsistent light conditions was solved in this study [112] by the use of an umbrella. This model was employed offline and displayed decreased performance because of differing training datasets from the data collected in the field and, therefore, highlighted the need to capture more images using mobile devices in typical field settings and to use those very images for the training of the models in order to improve the accuracy. In deploying open-source platforms and CNNs, there is an additional challenge in developing tools that can analyze and determine a wide variety of phenotypes from various crops, and it has not been possible to develop a one-size-fits-all platform for analysis. However, various platforms are thus far available for some phenotyping tasks.

## 4. Artificial Intelligence and Field Phenotyping

In order to screen plants for valuable traits (such as grain size, abiotic stress tolerance, product quality, or yield potential), experiments with repeated trials are required in different environments based on the objectives of the study. Much of the discussion of phenotyping has focused on the measurement of individual plants in controlled environments. However, controlled environments do not provide an accurate representation of plant growth in open-air conditions [7]. Field-based phenotyping (FBP) is now increasingly widely recognized as the only approach that gives accurate depictions of the traits in actual cropping systems. Currently, sensor systems suitable for high-throughput field phenotyping can simultaneously measure multiple plots and fuse a multitude of traits in different data formats [17]. Through the use of vehicles carrying multiple sets of sensors, FBP platforms are transforming the characterization of plant populations for genetic research and crop improvement. Accomplishing FBP in a timely and cost-effective manner has led to the use of unmanned aircraft, wheeled vehicles, or agricultural robots to deploy multiple sensors that can measure plant traits in brief time intervals [7]. Therefore, phenotyping in many crop breeding programs is now being conducted by combining instruments with novel technologies such as non-invasive imaging, robotics, and high-performance computing on the FBP platforms [8].

**Figure 4:**
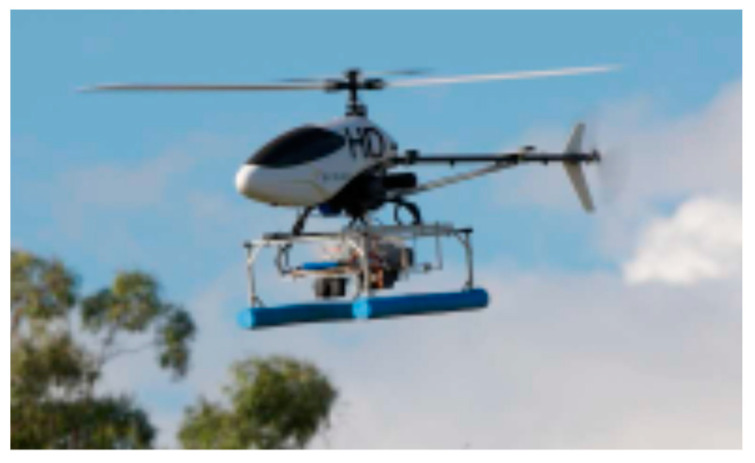
The CSIRO autonomous helicopter system. Adapted from Merz & Chapman, 2012 [113].

Unmanned aircraft are particularly attractive for data acquisition because they enable sensing with a high spatial and spectral resolution for a relatively low cost. Unmanned helicopters (such as the one shown in Figure 4) can carry various sensors and have the accommodation to carry larger sensors. In windy conditions, helicopters enable precise flight control and operations in cluttered environments because of their maneuverability and ability to fly at low speeds. Replicated collection of sensor data can be achieved through automatic flight control when the helicopter is equipped with algorithms for ground detection, obstacle detection and avoidance, and stable effective control [113]. Modern unmanned aerial systems (UAS) are better equipped to manage the harsh environmental conditions and obstacles due to rapid advances in technology such as collision technology, optical sensors for machine vision, GPS, accelerometers, gyroscopes, and compasses. However, they still face the challenge of limited battery power, with electric batteries providing between 10 to 30 min of battery power [25].

Although not commonly used for phenotyping, wheeled vehicles are sometimes used in phenotyping systems for some research projects since they also provide for proximal phenotyping. They have the advantage of being able to cover large areas and operate for longer periods, along with the disadvantage of compacting and damaging the soil and being costly because of the human labor required to operate the vehicle. According to White et al. 2012 [7], high-clearance tractors were expected to play a more central role in FBP as the wheeled vehicles of choice due to their high vertical clearance, availability, and ease of use. They can be used for continuous measurements at different stages of the crop growth process and can operate for longer periods compared to the UAVs. A variation of high clearance tractors in the form of mobile motorized platforms, which eliminate the need for human labor in the field, have been developed and tested in various phenotyping applications [114,115].

Phenotyping data collected using these FBP systems faces the challenge of instability during motion and weather changes, which cause occlusion and mal-alignment in the images. This is partially addressed by using proximal sensing at slower speeds in order to improve image resolution (although this limits the areal coverage in one flight and does not fully solve the misalignment). Data processing for this data has significantly improved in recent years with AI-enabled snapshot and line scanning imaging software, optimized specifically for unmanned aircraft such as structure-from-motion photogrammetry [87,116]. One approach that has been proposed to enhance the application of these platforms for sensing in field conditions is adapting field conditions to align with the HTP field techniques (rather than the traditional approach of adapting the instruments for the field), depending on the crop of interest without compromising the realistic crop evaluation in field conditions [6].

## 5. Phenotyping Communities and Facilities

The growth in plant phenotyping research coupled with the integration of AI technology has fostered the development of laboratories and centers equipped with high-throughput phenotyping technologies. Some of these plant phenotyping centers are members of the International Plant Phenotyping Network (IPPN), which works with various member organizations in academia and industry to distribute relevant information about plant phenotyping and increase its visibility [117]. Partner facilities such as the Australian Plant Phenomics Facility, a government-funded national facility, provide access to infrastructure such as glass-house automation technologies, digital imaging technologies, long-term data storage, etc. [118]. Such facilities have and continue to provide subsidized access to advanced AI-phenotyping technologies that would otherwise be inaccessible due to the costs of operation and maintenance. In addition, as high-throughput phenotyping becoming more common, there has come the issue of data-merging with many laboratories gathering phenotypic data that rarely enter the public domain where it could be accessed by other institutions to foster interdisciplinary research [8]. Networks such as the IPPN continue to provide access to phenotyping information generated by member organizations which is key in enabling cooperation between the organizations and advancing the phenomics agenda through collaborative research.

Besides the academic institutions and government organizations, private industry companies (a few of which have been highlighted here) are establishing themselves as key providers and facilitators of plant phenotyping AI technology around the world. Biopute technology provides high-end research instruments such as multispectral cameras for field phenotyping, drones for aerial photography, and provides after-sales support services to their customers. In partnership with universities and research institutes, Biopute provides innovations that are contributing to the progress of plant phenotyping in China (http://www.bjbiopute.cn, accessed on 1 June 2021). KeyGene is an agricultural biotechnology company providing tools for precision breeding and digital phenotyping investing in deep learning-based algorithms and virtual reality for data visualization (https://www.keygene.com, accessed on 1 June 2021). PhenoTrait Technology Co., Ltd. mainly focuses on plant phenotyping using the photosynthesis characteristics of plants and promoting the use of phenotyping technologies to improve crop quality, crop yield, and environmental conditions in China. Some of their products include high-throughput phenotyping instruments, chlorophyll fluorescence imaging systems, etc. (http://www.phenotrait.com, accessed on 1 June 2021). Photon Systems Instruments (PSI) is a company in the Czech Republic that also supplies a range of phenotyping systems, both field and laboratory-based, including root system phenotyping. They have also incorporated machine learning to integrate robotics into the systems they develop to better automate the processes (https://psi.cz, accessed 1 June 2021).

## 6. Conclusions

Recent advancements in high-throughput phenotyping technologies have led to significant strides in plant phenomics. The on-going integration of artificial intelligence into these technologies promises progression into smarter and much faster technologies with significantly lower input costs. In the area of phenotyping image data analysis, the integration of AI into the data management pipeline of tomography and thermography is on a lower scale in comparison to the other imaging techniques. The application of deep learning in the data analysis of these techniques is promising, as it has been successfully implemented in analysis of composite materials [88] and medical diagnostics [94]. As much as field phenotyping is the most effective way to collect phenotypic data, it is still being conducted on a relatively lower scale than is possible. Artificial intelligence technologies also require large amounts of data from various sources to improve their accuracy. This provides an opportunity to invest more into the tailoring of current technologies for field data collection and the utilization of already existing AI adaptable technologies, such as smartphones, to increase the quantity of quality data. Smartphones have become widespread consumer products, and the simplicity and ease of use of their sensors suggest that their use can be explored in agriculture [104]. Some of the challenges that would need to be addressed are that advanced signal processing on smartphones has to cope with constraints such as low battery life, restricted computational power, or limited bandwidth [104]. The use of citizen science alongside professional researchers [119] in data collection also has the potential to aid in increasing the amount of data collected. The overall goal of employing these approaches and technologies is to provide the infrastructure that allows for tracking how plant traits progress throughout the growing season and facilitate the coordination of data analysis, management, and utilization of results using AI methods.

## Figures and Tables

**Table 1:** Examples of ML-based approaches that have been applied in phenotyping tasks.

ML-Based Approach	Application	Plant	Reference
Bag-of-keypoints, SIFT	Identification of plant growth stage	Wheat	[41]
Decision tree	Plant image segmentation	Maize	[42]
SIFT, SVM	Taxonomic classification of leaf images	A group of varied genera and species	[43]
MLP, ANFIS	Classification	Wheat	[44,45]
kNN, SVM	Classification	Rice	[46]

Abbreviations: SIFT, Scale Invariant Features Transforms; kNN, k-nearest neighbor; SVM, Support Vector Machine; MLP, Multilayer Perceptron; ANFIS, Adaptive Neuro-fuzzy Inference System.

**Table 2:** Examples of deep learning architectures applied in plant phenotyping using transfer learning.

Deep Learning Architecture	Application	Plant	Reference
AlexNet, ZFNet, VGG-16, GoogLeNet, ResNet-50, ResNet-101, ResNetXt-101	Identification of biotic and abiotic stress	Tomato	[49]
VGG-16, VGG-19	Semantic segmentation of crops and weeds	Oilseed rape	[50]
Xception net, Inception-ResNet, DenseNet	Weed identification	Black Nightshade	[51]
GoogLeNet	Plant disease classification	A group of 12 plant species	[52]
VGG-16, VGG-19, Inception-v3, ResNt50	Classification of biotic stress	Apple	[53]
YOLOv3	Leaf counting	*Arabidopsis*	[54]

**Table 3:** Visualization techniques and applications [60].

Imaging Technique	Applications	Reference
Fluorescence	Photosynthesis features Metabolite composition Pathogen infection	[14,61,62,63]
RGB Imaging	Photosynthesis characteristics Pathogen infection Nutritional deficiencies	[15,22,64,65]
Thermography	Irrigation management Transpirational characteristics	[13,66,67,68]
Tomography	Tissue structure and metabolites Monitoring physiological and biochemical processes that occur in vivo	[69,70,71]
Spectroscopy	Identification of physiological responses, pathogens, and pests Surface structure growth and movements, pigment content	[16,72,73,74]

## Data Availability

No new data were created in this study. Data sharing is not applicable to this article.
